# Thianthrenium Salts in Photochemistry

**DOI:** 10.1021/acs.accounts.5c00863

**Published:** 2026-03-04

**Authors:** Zibo Bai, Tobias Ritter

**Affiliations:** 28314 Max-Planck-Institut für Kohlenforschung, D45470 Mülheim an der Ruhr, Germany

## Abstract

Thianthrenium (TT) salts have emerged as versatile reagents with utility across transition-metal (TM) catalysis, photochemistry, biocatalysis, electrochemistry, and polar transformations. Initially introduced as an aryl (pseudo)­halide surrogate in traditional TM-catalyzed cross-coupling reactions, thianthrenium salts have since demonstrated conceptually distinct advantages over their (pseudo)­halide analogues in single-electron mediated processes, particularly under visible-light irradiation.

The positively charged thianthrenium group raises the substrate’s reduction potential into the range accessible to common photocatalysts, and upon single-electron transfer, the exocyclic C–S_TT_ bond undergoes ultrafast mesolytic cleavage to generate aryl or alkyl radicals while avoiding the back-electron-transfer often observed with halide substrates. This combination of favorable redox properties and rapid bond fragmentation distinguishes thianthrenium salts as efficient radical precursors in photoredox catalysis.

Beyond the electron transfer mechanism in photoredox catalysis, thianthrenium salts have distinct advantages in *triplet energy transfer (EnT)* catalysis. In contrast to simple aryl (pseudo)­halides, which possess high triplet energies (*E*_T_ ≈ 78–82 kcal/mol), Ar–TT^+^ salts exhibit consistently lower triplet energies (*E*_T_ ≈ 60–66 kcal/mol), largely independent of the arene substitution pattern. This energy range allows for efficient *triplet–triplet energy transfer* from photosensitizers such as thioxanthone (TXO, *E*_T_ = 65.5 kcal/mol) for radical generation via EnT in high quantum yield.

In addition to photocatalytic pathways, direct photolysis of thianthrenium salts has emerged as a third mode of activation. This reactivity was initially employed to homolyze the CF_3_–S_TT_ bond of trifluoromethyl thianthrenium (CF_3_-TT^+^) triflate that has a low bond dissociation energy (BDE), with blue LEDs. A more general, biocompatible approach emerged through the development of selenonium-based TT analogues. TT-like selenonium-based reagents have been designed to realize *site-selective selenylation* of electron-rich aromatic residues on biomacromolecules in aqueous media. While the C–Se bond in these selenonium salts remains stable under ambient conditions, it undergoes efficient homolytic cleavage upon irradiation due to their visible light absorption and low BDE of ∼70 kcal/mol for the C–Se bond. Therefore, photochemical late-stage modifications of peptides, proteins, and nucleic acids can be achieved under physiologically compatible conditions.

This Account retraces the conceptual evolution of thianthrenium chemistry in our laboratory—from its origins in aromatic C–H functionalization to its diverse applications in photochemistry. We highlight conceptual and practical advances enabled by thianthrenium salts in photocatalysis, which are classified into two categories: photocatalytic SET (photoredox catalysis) and photocatalytic EnT. Within photoredox catalysis, three mechanistic modes are distinguished: (i) conventional photoredox catalysis; (ii) dual photoredox/transition-metal catalysis; and (iii) photoinduced transition-metal catalysis. In addition, we discuss the direct homolytic cleavage of thianthrenium and selenium salts under visible-light irradiations. By contrasting these strategies, we explain how thianthrenium chemistry provides practical and mechanistically distinct solutions to modern radical chemistry.

## Graphical abstract

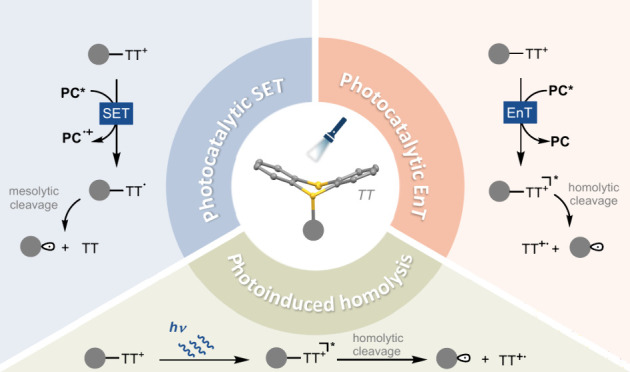

## Key References

- Berger, F.; Plutschack, M. B.; Riegger, J.; Yu, W.; Speicher, S.; Ho, M.; Frank, N.; Ritter, T. Site-selective and versatile aromatic C–H functionalization by thianthrenation. *Nature* **2019**, *567*, 223–228.^1^ A practical para-selective C–H thianthrenation protocol was developed to access aryl thianthrenium salts with broad functional group tolerance. The preliminary results demonstrated the redox reactivity of aryl thianthrenium salts under photoredox conditions.
- Li, J.; Chen, J.; Sang, R.; Ham, W.-S.; Plutschack, M. B.; Berger, F.; Chabbra, S.; Schnegg, A.; Genicot, C.; Ritter, T. Photoredox catalysis with aryl sulfonium salts enables site-selective late-stage fluorination. *Nat. Chem*. **2020**, *12*, 56–62.^2^ The conceptual advantages of aryl thianthrenium salts over aryl (pseudo)­halides in photoredox catalysis was demonstrated for the first time, enabling site-selective late-stage fluorination of complex arenes under mild conditions.
- Cai, Y.; Roy, T. K.; Zähringer, T. J. B.; Lansbergen, B.; Kerzig, C.; Ritter, T. Arylthianthrenium Salts for Triplet Energy Transfer Catalysis. *J. Am. Chem. Soc*. **2024**, *146*, 30474–30482.^3^ Aryl thianthrenium salts were shown to enable efficient triplet energy transfer catalysis, facilitating aryl–heteroatom difunctionalization of alkenes.
- Lin, S.; Hirao, M.; Hartmann, P.; Leutzsch, M.; Sterling, M. S.; Vetere, A.; Klimmek, S.; Hinrichs, H.; Mengeler, J. M.; Lehmann, J.; Samsonowicz-Górski, J.; Berger, F.; Ritter, T. A selenoxide for single-atom protein modification of tyrosine residues enabled by water-resistant chalcogen and hydrogen bonding. *Nat. Chem*. **2025**, *17*, 1331–1339.^4^ A rationally designed selenoxide enabled site-selective C–H functionalization of tyrosine residues of peptides and proteins in aqueous media. The resulting selenonium linchpin facilitated small structural modifications of biomacromolecules through direct photolysis.

## 1 Introduction

The generation of carbon-centered radicals under photochemical conditions provides a powerful strategy for constructing C–C and C–X bonds under mild conditions.^5–9^ However, selection of an appropriate radical precursor remains a central challenge, as the trade-offs between redox potential, stability, and fragmentation kinetics define the practical limits of radical chemistry. Over the past decades, a wide set of carbon-centered radical precursors has been developed, particularly for the generation of alkyl and aryl radicals. Representative alkyl radical precursors include sodium sulfinate salts,^10^ trifluoroborates,^11^ redox-active esters,^12^ Katritzky salts,^13^ Hantzsch esters,^14^ and hypervalent bis-catecholato silicon compounds.^15^ For aryl radicals, commonly used precursors include aryl diazonium salts,^16,17^ diaryliodonium salts,^18^ boronic acids,^19^ sulfonyl chlorides,^20^ and sulfonium salts.^21^ Reagents that can serve as both alkyl and aryl radical sources include carboxylic acids,^22^ (pseudo)­halides,^7,23,24^ and thianthrenium salts.^25,26^ Among these, thianthrenium salts demonstrate fundamentally distinct properties when compared to the other reagents.^23,26–28^ Early synthetic routes to aryl radical species largely depended on stoichiometric radical initiators or reductants, such as trialkyltin hydrides for aryl halides,^30^ or transition-metal reagents^31^ for the reduction of diazonium salts. Recent progress in redox catalysis, particularly under irradiation,^5–9^ has renewed attention to aryl radical processes.^23,26–28^ Photocatalysis can generate aryl radicals through single-electron transfer (SET) or energy transfer (EnT) pathways from a variety of precursors, most notably aryl halides,^23^ diazonium salts,^17^ sulfonium salts,^21^ and diaryliodonium salts.^18^ Aryl halides are among the most accessible and stable precursors but their negative reduction potentials typically fall below −2.0 V vs SCE, which renders activation difficult with common photoredox catalysts and requires the use of well-designed strongly reducing photocatalysts or multiphoton strategies such as consecutive photoinduced electron transfer (conPET).^23^ In contrast, aryl diazonium salts, as one of the earliest aryl radical precursors, are readily reduced via SET (*E*_1/2_ = ∼0 V vs SCE) to generate aryl radicals rapidly through nitrogen extrusion.^17^ However, diazonium salts suffer from poor thermal stability, with some even being explosive.^32^ Diaryliodonium salts, on the other hand, offer a balance between stability and redox accessibility. As bench-stable crystalline solids with reduction potentials in the range of −0.4 to −0.8 V vs SCE, they can be efficiently reduced by common photocatalysts.^18^ Upon reduction, they release aryl radicals efficiently but produce stoichiometric aryl iodide byproducts. Moreover, diaryliodonium salts are light-sensitive and their preparation typically requires strong oxidants, which limits substrate scope.^18^ Simple aryl (pseudo)­halides like phenyl halides have higher triplet energies on the order of *E*_T_ = 78–82 kcal/mol and most common photocatalysts (*E*_T_ < 66 kcal/mol) therefore cannot activate them via EnT catalysis,^33^ while aryl diazonium and iodonium salts mainly serve as electron acceptors rather than energy acceptors due to their high, positive reduction potentials.^3^

In contrast, thianthrenium salts demonstrate several features rarely coexisting in other precursors: (1) thianthrenium salts display bench stability, allowing convenient handling and storage, in contrast to diazonium and iodonium salts.^1^ (2) Their reduction potential of ≈ −1.5 V vs SCE is well matched to commonly used photocatalysts, which enables efficient SET activation with visible light.^2^ (3) Upon reduction, thianthrenium salts undergo rapid C–S_TT_ bond fragmentation to release radicals,^2^ while bypassing back-electron transfer (BET) pathways that hamper other aryl (pseudo)­halides.^23,34^ (4) Ar–TT^+^ salts possess triplet energies of 60–66 kcal/mol,^3^ largely independent of aromatic substitution, allowing efficient EnT processes with photosensitizers such as TXO. These combined features distinguish thianthrenium salts as a practical and general platform for photocatalytic radical generation (Figure 1).

**Figure 1:**
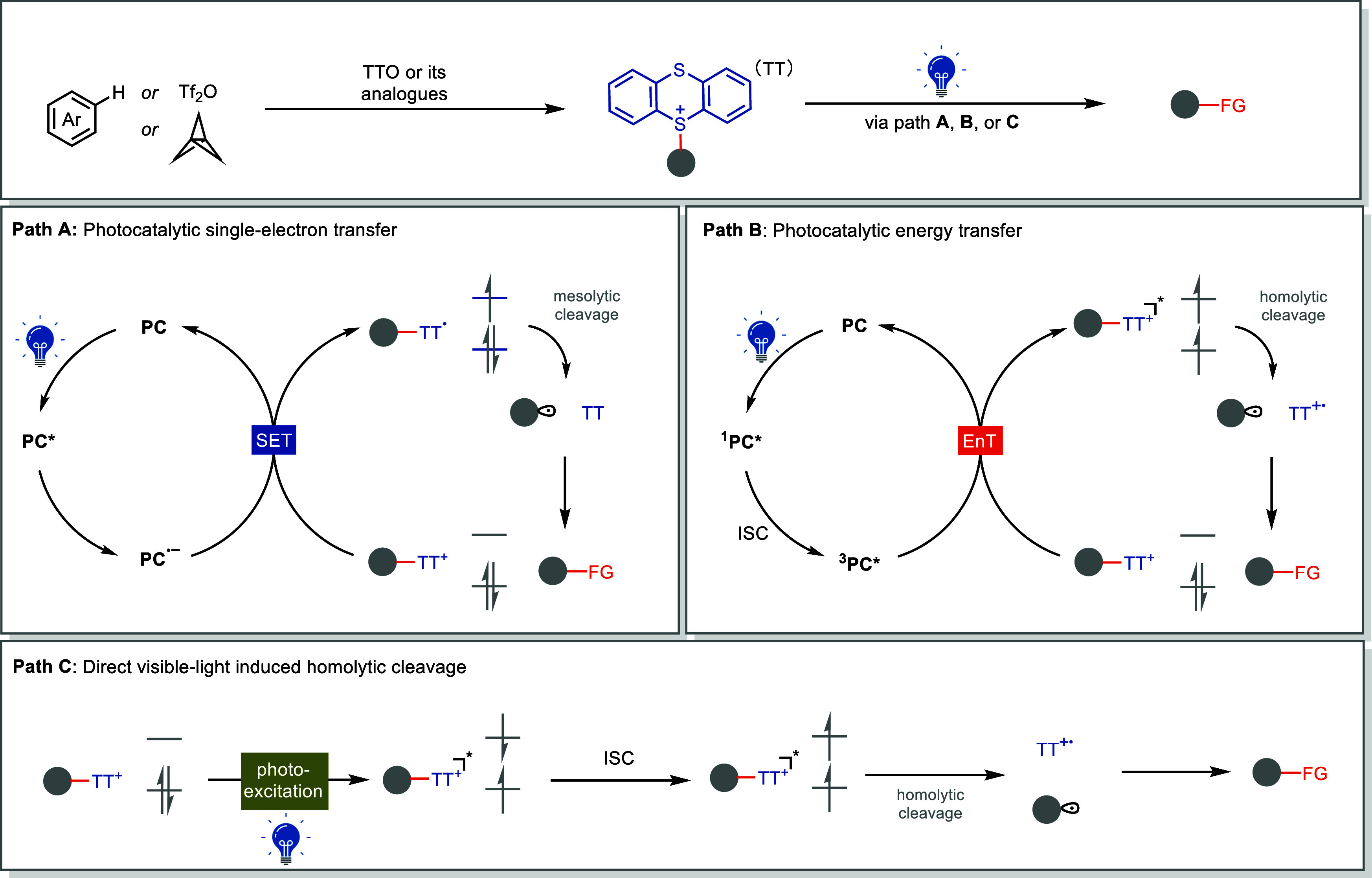
Our achievements in photochemical functionalization enabled by thianthrenium salts. Tf_2_O, trifluoromethanesulfonic anhydride; PC, photocatalyst; ISC, intersystem crossing.

Beyond photocatalytic activation, radicals can also be generated from thianthrenium salts through direct photoinduced homolysis^35–37^ or electron donor–acceptor (EDA) complex formation.^38,39^ While most thianthrenium salts do not absorb visible light and typically require UV irradiation for direct photolysis,^40^ an exception is the trifluoromethyl thianthrenium salt (CF_3_–TT^+^),^35–37^ which undergoes homolytic cleavage with blue or purple light. This behavior likely arises from its relatively low BDE of the S_TT_–CF_3_ bond (35.8 kcal/mol).^35,41^ Likewise, the lower BDE of the Se–C bond also allows for direct homolysis to generate synthetically useful radicals. Rationally designed selenoxides similar to the TT chemistry enable site-selective selenylation of aromatic residues in proteins^4^ and nucleic acids.^42^ The resulting selenonium salts absorb visible light and homolysis of the weak C–Se bond, e.g., BDE ≈ 70 kcal/mol for C–Se in tyrosine conjugates allows for late-stage modification of biomacromolecules.

Reaction chemistry with arylthianthrenium salts has only been introduced in 2019, when other sulfonium salts already had established utility. While simple aryldimethylsulfonium salts have quite distinct reactivity and also result in thioether byproducts that are more Lewis acidic than TT, which may reduce catalyst activity,^43^ other cyclic triaryl sulfonium reagents such as dibenzothiophenium (DBT) and phenoxathiinium (PHT) salts may in part exhibit similar stereoelectronic properties and reactivities as to TT-based salts.^21,44,45^ Its generality in installation and transformation often makes the thianthrene substituent a promising initial choice but other sulfonium-based salts can be advantageous in specific cases.

## 2 Discovery of Thianthrenium Salts

Our exploration of thianthrenium chemistry began with a persistent challenge in aromatic C–H functionalization: achieving high regioselectivity without sacrificing reactivity. Early work in our group focused on direct arene fluorination,^46^ but most substrates afforded mixtures of constitutional isomers, revealing a conceptual limitation: electronically unbiased arenes lacked intrinsic elements capable of controlling site selectivity.

We reasoned that a more general solution would require introducing a versatile “linchpin” substituent that could not only realize regioselectivity but also serve as a platform for downstream diversifications. In 2016, we reported a well-defined palladium catalyst that promotes the reaction of Selectfluor with arenes to afford arylammonium salts via a highly regioselective radical substitution process (Figure 2A).^47^ Yet, the ammonium substituent proved synthetically inflexible, serving more as a terminus than as a platform.

**Figure 2:**
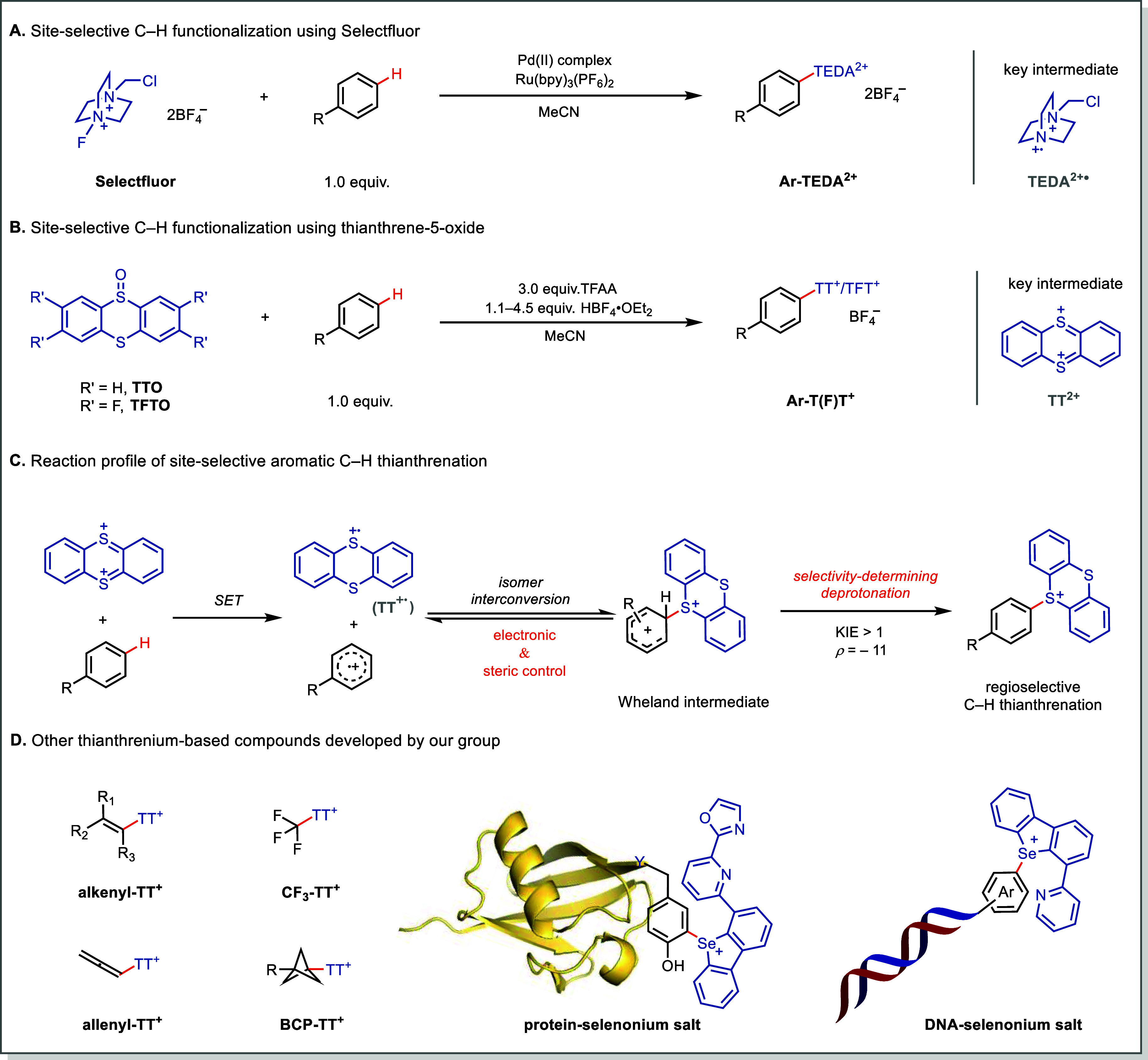
Discovery of thianthrenium salts. (A) Site-selective C–H functionalization using Selectfluor. (B) Site-selective C–H functionalization using thianthrene-5-oxide. (C) Reaction profile of site-selective aromatic C–H thianthrenation. (D) Other thianthrenium-related reagents developed by our group. TFAA, trifluoroacetic anhydride; KIE, kinetic isotope effect.

We therefore sought a reagent capable to distinguish subtle electronic effects to achieve site selectivity while remaining synthetically versatile. In 2019, our group reported novel and bench-stable thianthrene-5-oxide (TTO) and its fluorinated analogue tetrafluorothianthrene-5-oxide (TFTO), which can functionalize a broad scope of electron-rich and -neutral arenes with high site-selectivity (Figure 2B).^1^ Mechanistic studies revealed that the high para selectivity did not arise from the thianthrene dication (TT^2+^) addition itself, but from a reversible formation of Wheland intermediates.^48^ The observed selectivity reflects the relative stabilities of the three constitutional Wheland intermediates in monosubstituted arenes, where electronic effects favor para over meta substitution and steric effects disfavor ortho attack (Figure 2C). Since then, the thianthrenium platform has been extended to alkenylation,^49,50^ allenylation,^51^ trifluoromethylation,^35^ bicyclo[1.1.1]­pentylation,^37^ and biomacromolecular functionalization using its selenonium analogues^4,42^ (Figure 2D).

## 3 Thianthrenium Salts in Photoredox Catalysis

### 3.1 Conceptual Advantages of Thianthrenium Salts in Photoredox Catalysis

Thianthrenium salts were initially employed as surrogates for aryl (pseudo)­halides in transition-metal-catalyzed cross-coupling reactions such as Suzuki, Negishi, and Heck couplings.^1^ In the context of photoredox catalysis, however, these salts display a distinct electronic and kinetic profile that fundamentally distinguishes them from conventional aryl (pseudo)­halides.^2^ Owing to the positive charge of the thianthrenium group, Ar–TT^+^ salts exhibit less negative reduction potentials than their aryl halide counterparts with identical substituents, enabling their activation within the operating window of conventional photocatalysts (≥1.7 V vs SCE).^5^ A correlation between the reduction potential and the mesolytic cleavage rate of aryl halides and aryl thianthrenium salts (Ar–TT^+^) highlights how the thianthrenium scaffold uniquely couples redox accessibility with rapid bond fragmentation (Figure 3A). In contrast, aryl halides are either too difficult to reduce (typically < −2.0 V vs SCE) or fragment too slowly, limiting efficient aryl-radical formation under photoredox conditions.

**Figure 3:**
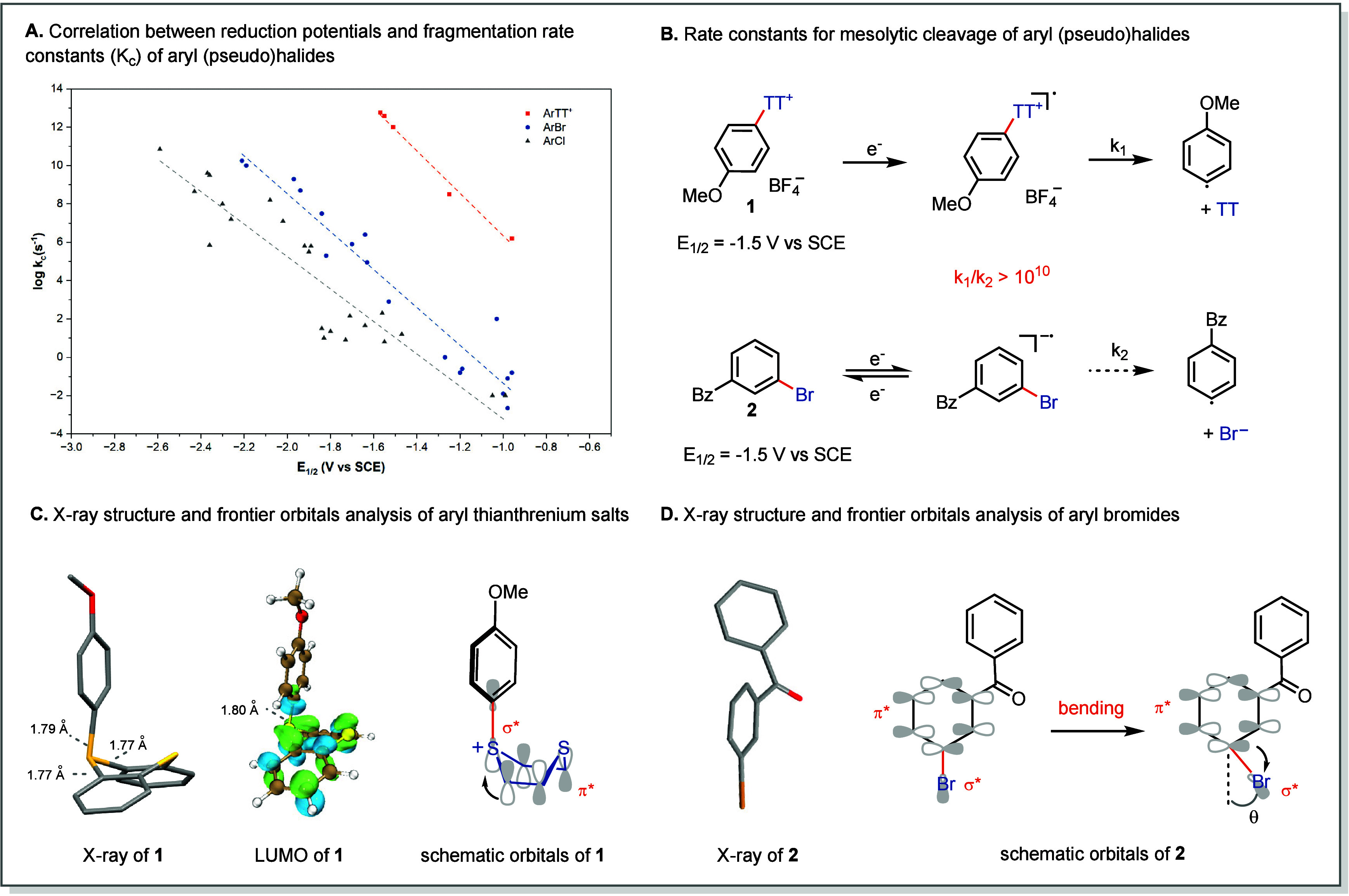
Conceptual advantage of aryl thianthrenium salts in photoredox catalysis. (A) Correlation between reduction potentials and fragmentation rate constants (Kc) of aryl (pseudo)­halides. (B) Rate constants for mesolytic cleavage of aryl (pseudo)­halides of **1** and **2**. (C) X-ray structure and frontier orbitals analysis of **1** (CCDC: 1900278). (D) X-ray structure and frontier orbital analysis of **2** (CCDC: 838060).

The kinetic advantage of Ar–TT^+^ is particularly evident when comparing bond fragmentation rates following SET reduction. In aryl (pseudo)­halides, radical generation depends not only on the reduction event but also on how the C–X bond cleaves.^23^ Aryl iodides, with weaker C–I bonds, can undergo a concerted electron-transfer/bond-breaking process, while aryl bromides and chlorides typically follow a slower, stepwise pathway in which bond dissociation occurs after initial reduction.^52^ This stepwise mechanism allows back-electron transfer to compete with productive cleavage—a key limitation in photoinduced electron-transfer reactions involving aryl halides.^23,34,52^ By contrast, after SET reduction, the exocyclic C–S bond in Ar–TT^+^ can fragment nearly 10 orders of magnitude faster than the C–Br bond of aryl bromides with comparable redox potentials (Figure 3b).^2^

Although three distinct C–S bonds are present within aryl TT salts, only the exocyclic aryl C–S bond undergoes efficient fragmentation. Structural studies provide a clear rationale: X-ray crystallography of Ar–TT^+^ shows that the appended aryl substituent adopts a flagpole conformation relative to the dithiine boat conformation, and the exocyclic C–S bond (1.79 Å) is consistently longer compared to the two endocyclic bonds (1.77 Å) (Figure 3C, left).^2^ This geometric bias is further supported by density functional theory (DFT) calculations, which predict an exocyclic C–S bond length of 1.80 Å (Figure 3C, middle). After single-electron reduction, the exocyclic C–S bond elongates from 1.80 to 1.85 Å, providing a structural rationale for its selective cleavage while leaving the endocyclic C–S bonds intact.

The faster fragmentation of Ar–TT radical species relative to aryl bromide radical anions can be attributed to the stereoelectronic preorganization evident from the lowest unoccupied molecular orbital (LUMO) of ArTT^+^, which displays σ* antibonding character along the exocyclic C–S bond and favorable alignment with the π* system of TT (Figure 3C, middle and right). Accordingly, population of this orbital upon one-electron reduction directly weakens the exocyclic C–S bond and facilitates homolytic cleavage to generate aryl radicals. In contrast, for aryl bromide radical anions the C–Br σ* orbital is orthogonal to the arene π system, necessitating substantial geometric distortion before dissociation can occur (Figure 3D).^53,54^

### 3.2 Different Reaction Modes of Thianthrenium Salts in Photoredox Catalysis

The high reduction potential and ultrafast fragment rate upon SET reduction of aryl thianthrenium salts allow them to engage efficiently in diverse photoredox pathways. Depending on how the radical is generated and which species captures it afterward, we identified three representative modes that showcase the versatility of thianthrenium chemistry in photoredox catalysis (Figure 4): (1) photoredox catalysis, where the photocatalyst alone drives radical formation and coupling; (2) dual photoredox/transition-metal catalysis, where photochemical SET and organometallic bond construction cooperate in tandem; (3) photoinduced transition-metal catalysis, in which the metal complex itself acts as the photoactive species and mediates the bond formation steps.

**Figure 4:**
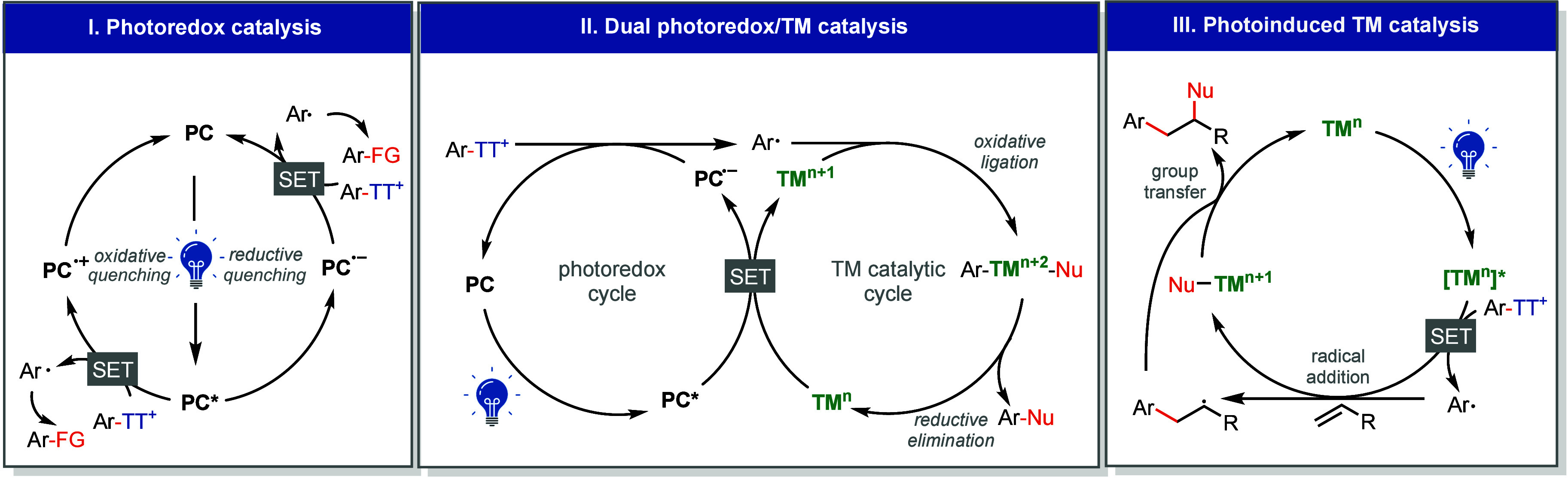
Different reaction modes of thianthrenium salts in photoredox catalysis.

#### 3.2.1 Photoredox Catalysis

Photocatalysts can reduce Ar–TT^+^ through SET, releasing an aryl radical that subsequently reacts with an external radical trap or coupling partner such as an olefin, heteroatom donor, or radical-transfer reagent. Representative transformations include borylation (**3**), phosphination (**4**), and Minisci-type arylation (**5**) reactions (Figure 5).^1^ The substrate scope achievable with Ar–TT^+^ salts extends well beyond that of conventional aryl (pseudo)­halides. In particular, electron-rich aryl radicals, which are not readily accessible through photochemical reduction of aryl halides, can now be generated smoothly.^23^ Moreover, the molecular complexity and late-stage functionalization attainable with Ar–TT^+^ surpass those of diazonium or iodonium precursors, owing to their bench stability, mild preparation, and predictable site selectivity.^27,28^

**Figure 5:**
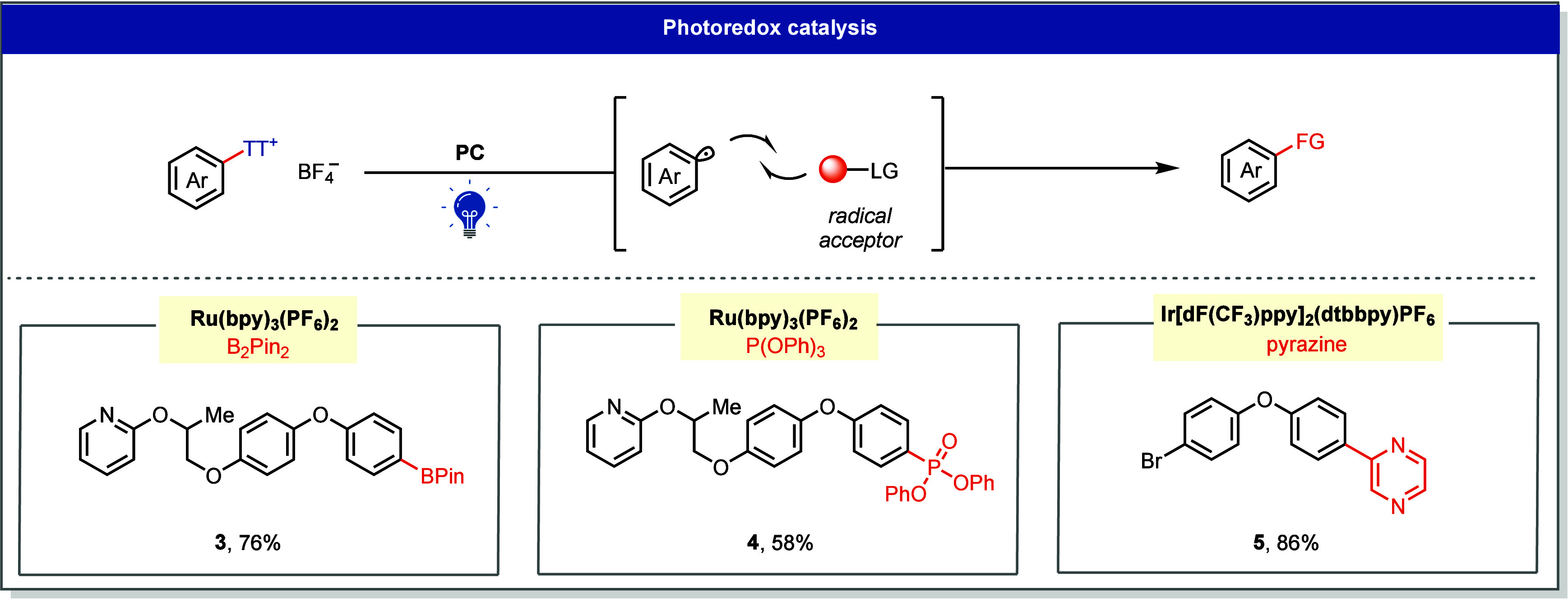
Photoredox-catalyzed borylation, phosphonation, and Minisci-type reaction of aryl thianthrenium salts. LG, leaving group.

Building on the success of two-component coupling reactions, we switch our attention to radical multicomponent coupling reactions using Ar-TT^+^ salts. A representative demonstration of this mechanistic mode is the *Meerwein-type bromoarylation of alkenes* under visible-light photoredox catalysis (Figure 6).^55^ In this transformation, a *N*-phenyl-benzo­[*b*]­phenothiazine (PTH) mediates oxidative quenching (*E*_1/2_ = −1.92 V vs SCE) to generate aryl radicals from Ar–TT^+^ via rapid C–S bond fragmentation (Figure 6A). The resulting nucleophilic aryl radical undergoes Giese-type addition to electron-poor alkenes to form a stabilized carbon-centered radical intermediate,^56^ followed by Br atom transfer from bromine to deliver α-bromo-substituted adducts (Figure 6B and 6C). Unlike conventional Cu-catalyzed Meerwein arylation reactions that rely on thermally unstable diazonium salts and often suffer from Sandmeyer-type halogenation side reactions, where halogen-atom transfer rate from CuX_2_ to an aryl radical occurs at *k* ≈ 10^8^ M^–1^ s^–1^ and thus competes with aryl radical addition to alkenes (activated alkenes: *k* ≈ 10^8^ M^–1^ s^–1^; unactivated alkenes: *k* ≈ 10^7^ M^–1^ s^–1^), the photoredox protocol using Ar–TT^+^ proceeds cleanly with broad functional-group tolerance.^55,57^

**Figure 6:**
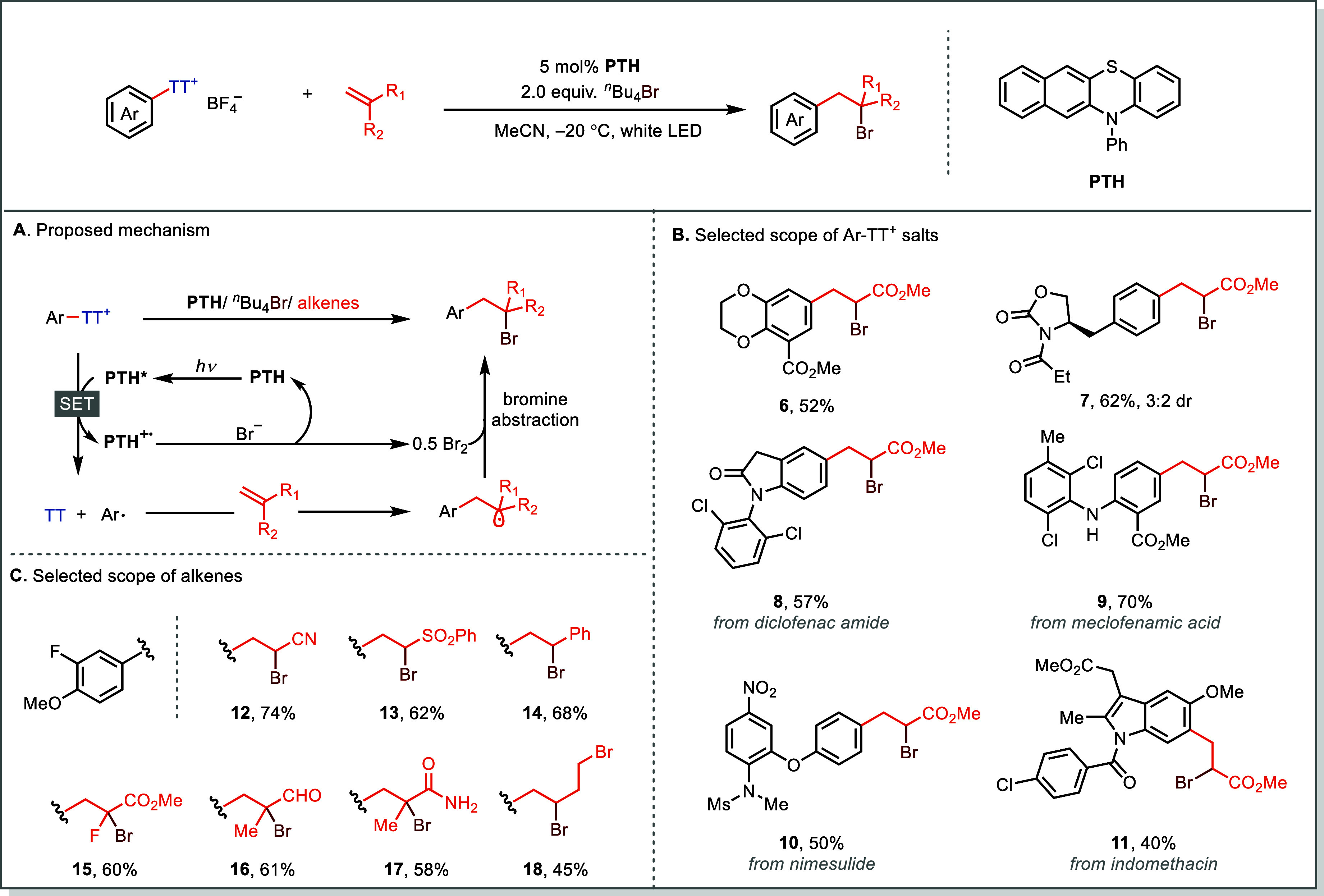
Photoredox-catalyzed Meerwein-type bromoarylation with arylthianthrenium salts.

#### 3.2.2 Dual Photoredox/Transition-Metal Catalysis

In photoredox catalysis, bond formation relies largely on the intrinsic polarity and reactivity of the radical and its acceptor. As a result, transformations are confined to substrates whose innate electronic properties favor productive coupling. Merging photoredox and transition-metal catalysis expands the synthetic scope further, linking outer-sphere photoinduced electron transfer with inner-sphere metal-mediated bond formation. In these cooperative systems, the photocatalyst modulates electron transfer while the TM executes C–C or C–heteroatom coupling challenging for thermally driven reactions.^5^

A key challenge in this system is the intrinsic redox mismatch between the photochemical radical-generation step and the rapid oxidative capture by the TM center. Thianthrenium salts proved to be ideal reagents for this system: their high reduction potentials and fast C–S bond fragmentation provide efficient radical generation while maintaining compatibility with high-valent metal intermediates. This concept was demonstrated in a series of photoredox/Cu-catalyzed overall redox-neutral aromatic transformations, including thiotrifluoromethylation (**19**),^1^ halogenation (**20**, **21**),^1^ cyanation (**22**),^1^ and trifluoromethylation (**23**) reactions,^58^ all proceeding with visible light (Figure 7).

**Figure 7:**
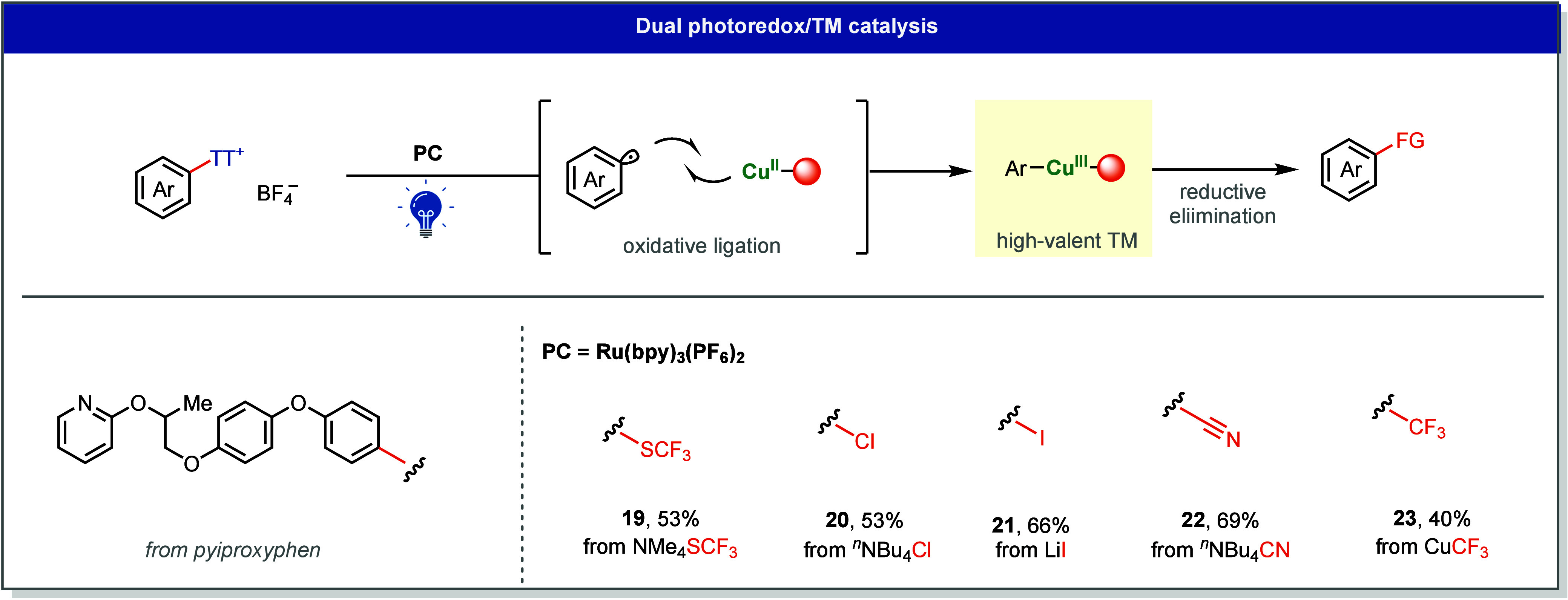
Dual photoredox/Cu-catalyzed reactions.

A remarkable example is the C­(*sp* ^2^)–F bond formation.^2^ In contrast to previous TM-catalyzed fluorination strategies that require functionalized aryl–metal species^59^ or high reaction temperature,^60,61^ the Ir/Cu dual-catalyzed fluorination of Ar–TT^+^ proceeds with visible-light and even tolerates protic and coordinating groups such as hydroxyl groups and amides (Figure 8A). An additional practical advantage arises from purification. Direct fluorination of aryl halides often complicates purification of the fluorinated product, especially when incomplete conversion leaves unreacted arylhalides.^60^ By contrast, the high polarity of Ar–TT^+^ salts ensures that any unreacted starting material is easily separated, enabling the access to analytically pure fluorinated products. A Stern–Volmer analysis revealed that the excited-state Ir­(III)* species (*E*_1/2_(*Ir^3+^/Ir^2+^) = +1.21 V vs SCE) undergoes reductive quenching by thianthrene (*E*_1/2_(TT^•+^/TT) = +1.26 V), generating Ir­(II), which subsequently reduces Ar–TT^+^ (*E*_1/2_ ≈ −1.5 V vs SCE) to produce the aryl radical.^2^ Electron paramagnetic resonance (EPR) spectroscopy detected both TT^•+^ and Cu­(II) species, consistent with a dual catalytic cycle where photoinduced SET and metal-mediated C–F bond formation proceed at the same time (Figure 8B). Radical trapping and clock experiments support further the formation of aryl radical under the standard conditions (Figure 8C). Unlike other leaving groups, TT possesses unique redox flexibility, serving as an electronic mediator that accelerates electron transfer between the photocatalyst and the Cu catalyst in this reaction.

**Figure 8:**
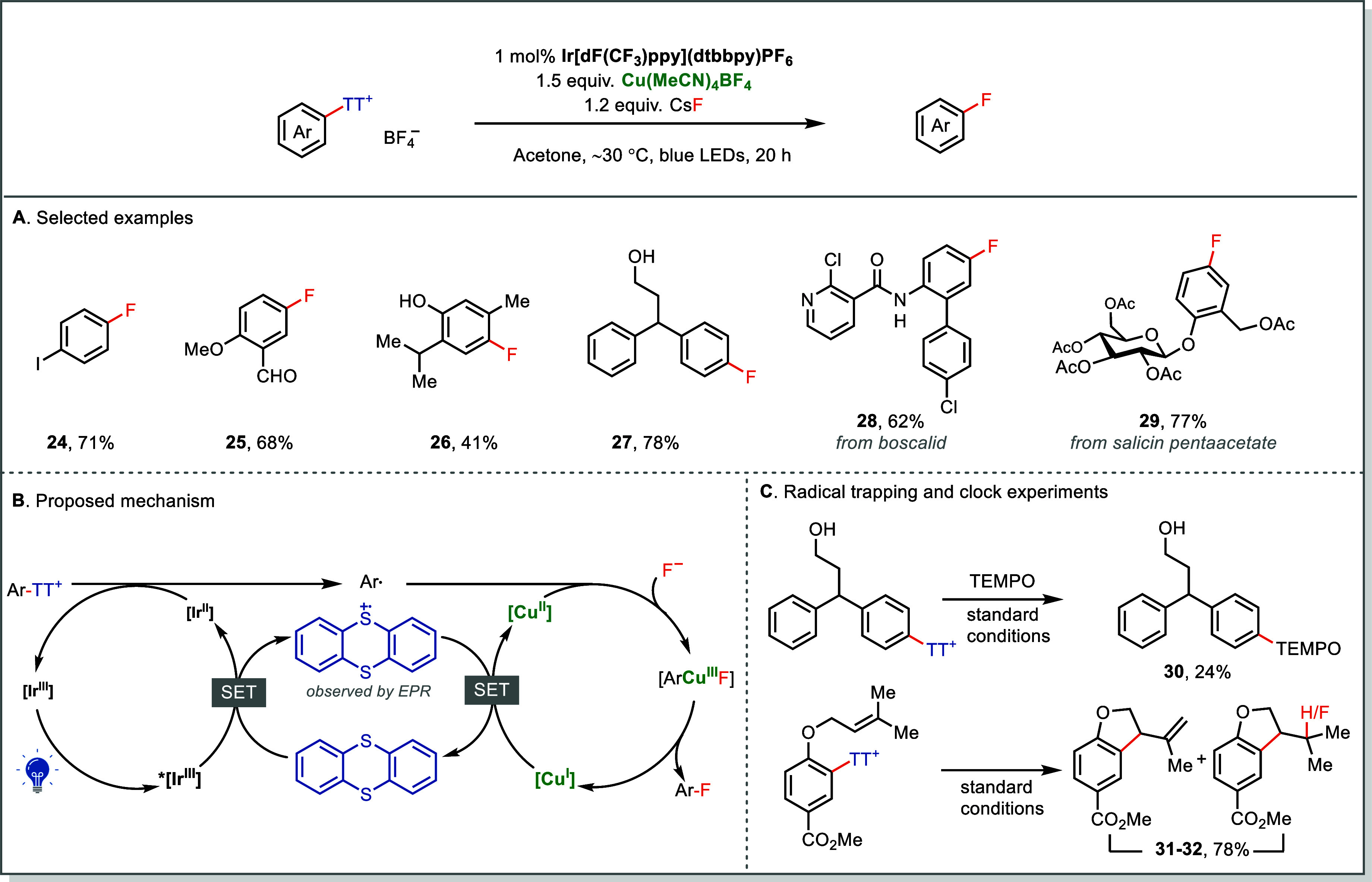
Dual photoredox/Cu-catalyzed fluorination of Ar-TT^+^ salts.

Analogous Cu-based systems mediate C–N and C–O couplings through the same mechanistic pathway, where photoreduction of Ar–TT^+^ generates aryl radicals that are oxidatively captured by Cu­(II)–Nu species (Nu = amine, hydroxide, or alkoxide) to form high-valent Cu­(III)–aryl intermediates, which subsequently undergo reductive elimination to yield the coupling products. In the C–N coupling variant, a broad range of nitrogen nucleophiles, including aliphatic amines, amides, and azoles, can be efficiently arylated, enabling late-stage diversification of complex molecules (Figure 9A).^62^ The complementary C–O and C–S coupling proceeds similarly, using water, phenols, alcohols, thiophenol, and thiols as nucleophiles to deliver phenols (**38**), aryl ethers (**39**–**41**), and aryl sulfides (**42**, **43**), respectively (Figure 9B).^63^

**Figure 9:**
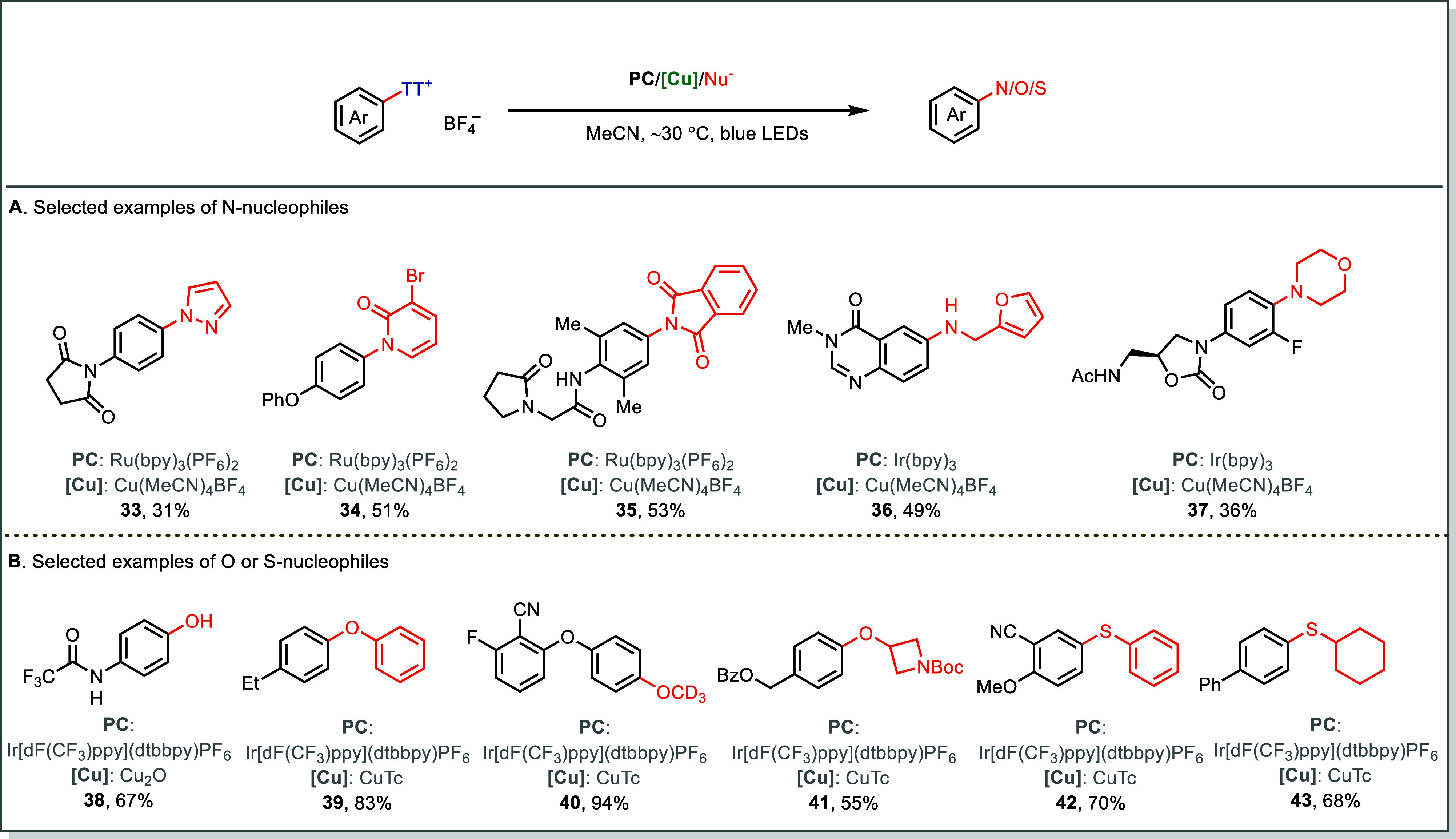
Dual photoredox/Cu-catalyzed amination, oxygenation, and thioetherification of Ar-TT^+^ salts.

Given that the electron-deficient nature of thianthrenium salts arises primarily from the cationic charge, the advantages (high reduction potential and rapid mesolytic cleavage) of Ar–TT^+^ in photoredox catalysis extend to alkyl thianthrenium salts as well.^25,26^ Our group has developed practical *O*-, *N*-, and *C*-alkylation reactions using stable BCP-based thianthrenium salts, thereby expanding the scope of bicyclopentylation beyond that accessible with any other reagent, including [1.1.1]­propellane (Figure 10A).^37^ The weak exocyclic C–S bond undergoes selective mesolytic cleavage upon single-electron reduction, generating BCP radicals that efficiently participate in Cu-catalyzed C–O, C–N, and Ni-catalyzed C–C bond formations.^37,41^ Notably, these transformations can be performed at a late stage and tolerate a wide range of functional groups, enabling installation of the BCP motif in complex molecules. This synthetic utility is illustrated by the concise preparation of BCP analogues of known pharmaceuticals, such as fluoxetine (**48**) (Figure 10B).

**Figure 10:**
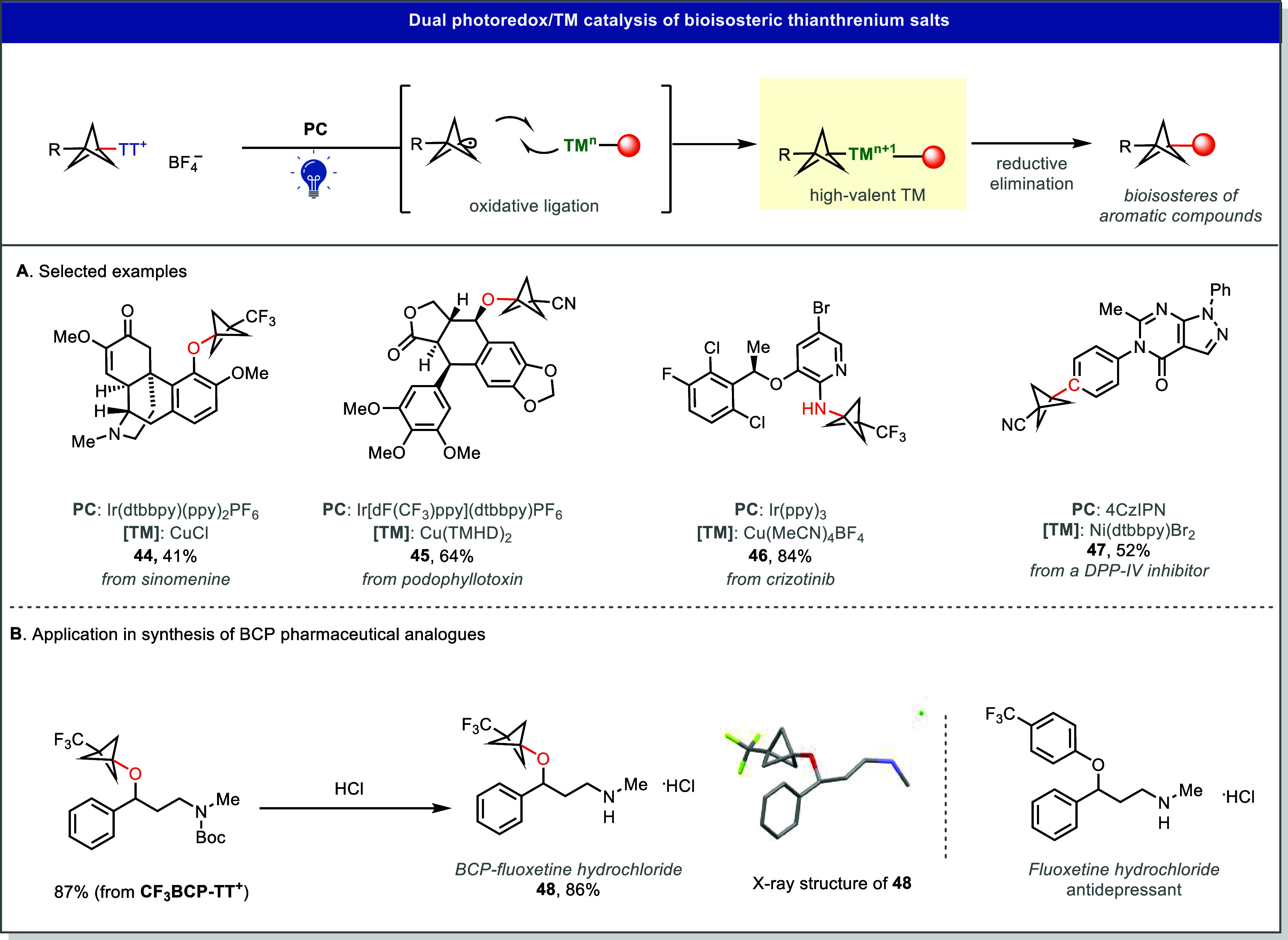
Dual photoredox/Cu or Ni-catalyzed *O*, *N*, *C*- bicyclopentylation using thianthrenium salts. X-ray of **48** (CCDC: 2286412). TMHD, 2,2,6,6-tetramethyl-3,5-heptanedionate.

#### 3.2.3 Photoinduced Transition-Metal Catalysis

A third reactivity mode of photoredox catalysis arises when the metal complex itself absorbs light and becomes the redox-active species. Photoexcitation promotes the metal complex into a charge-transfer excited state that transiently reorganizes its electronic structure and expands its redox window.^64^ The resulting excited species can engage in direct single-electron transfer with bound or nearby substrates, initiating catalytic cycles that are unattainable under ground state catalysis. This direct photochemical activation bypasses the outer-sphere redox mediation pathway of classical photoredox catalysis, providing access to high-energy intermediates and extending the redox reach of the transition metal catalyst.^64^ To make such photoexcited metal states productively, the substrate must match the redox and kinetic profile of the excited TM catalyst. Within this framework, Ar–TT^+^ serve as ideal partners: their high and uniform reduction potentials, coupled with fast and irreversible C–S bond fragmentation, enable efficient single-electron transfer and rapid generation of aryl radicals with visible light. These attributes allow photoexcited Cu or Ni complexes to engage in efficient radical generation and subsequent TM-mediated bond constructions.^65,66^

In the photoinduced Cu-catalyzed azidoarylation of alkenes, an *in situ*-formed *rac*-BINAP–Cu­(I)–azide complex acts as both photoredox and group-transfer catalyst (Figure 11).^65^ Upon visible-light excitation, the Cu­(I) complex A* reduces Ar–TT^+^ to form aryl radicals, which add to alkenes and are intercepted by Cu­(II)–azide species **B** to forge C–N_3_ bonds. This self-sustained photochemical cycle merges light absorption, radical generation, and bond construction within a single Cu catalytic cycle.

**Figure 11:**
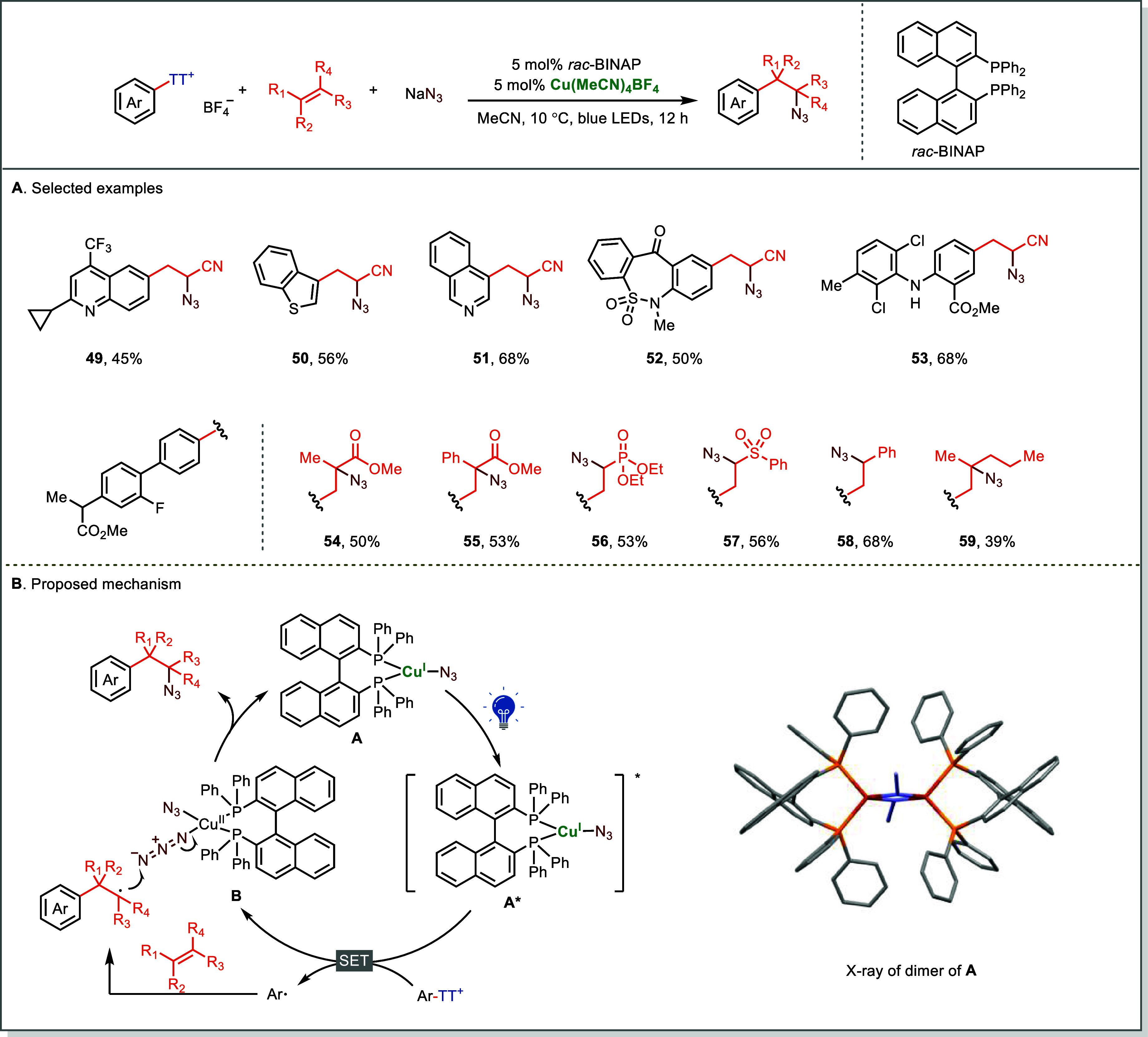
Photoinduced copper-catalyzed late-stage azidoarylation of alkenes via arylthianthrenium salts. X-ray of dimer of **A** (CCDC: 2247825).

Complementary reactivity arises in the Ni-catalyzed C–heteroatom coupling of Ar–TT^+^ salts, where irradiation of a NiCl_2_–amine complex **C** induces ligand-to-metal charge transfer (LMCT) to form an amine radical cation together with an active Ni­(I) species **D** (Figure 12A). The resulting Ni­(I) complex can reduce Ar–TT^+^ salts, generating high-valent Ni­(III) intermediates **E** via an in-cage rebound pathway (Figure 12B).^66^ The process can use simple nickel salts as catalysts at room temperature, and make C–N, C–O, C–S, and C–X bonds even with electron-rich arenes that are otherwise often unreactive using their halide analogues. The involvement of Ni­(I) species was substantiated: treatment of substrates with Ni­(COD)_2_ and FeCp_2_BAr^F^ delivered product in 90% yield (Figure 12C, entry 1). In contrast, neither Ni(0) nor Ni­(II) generated any product without irradiation (Figure 12C, entry 2 and 3).

**Figure 12:**
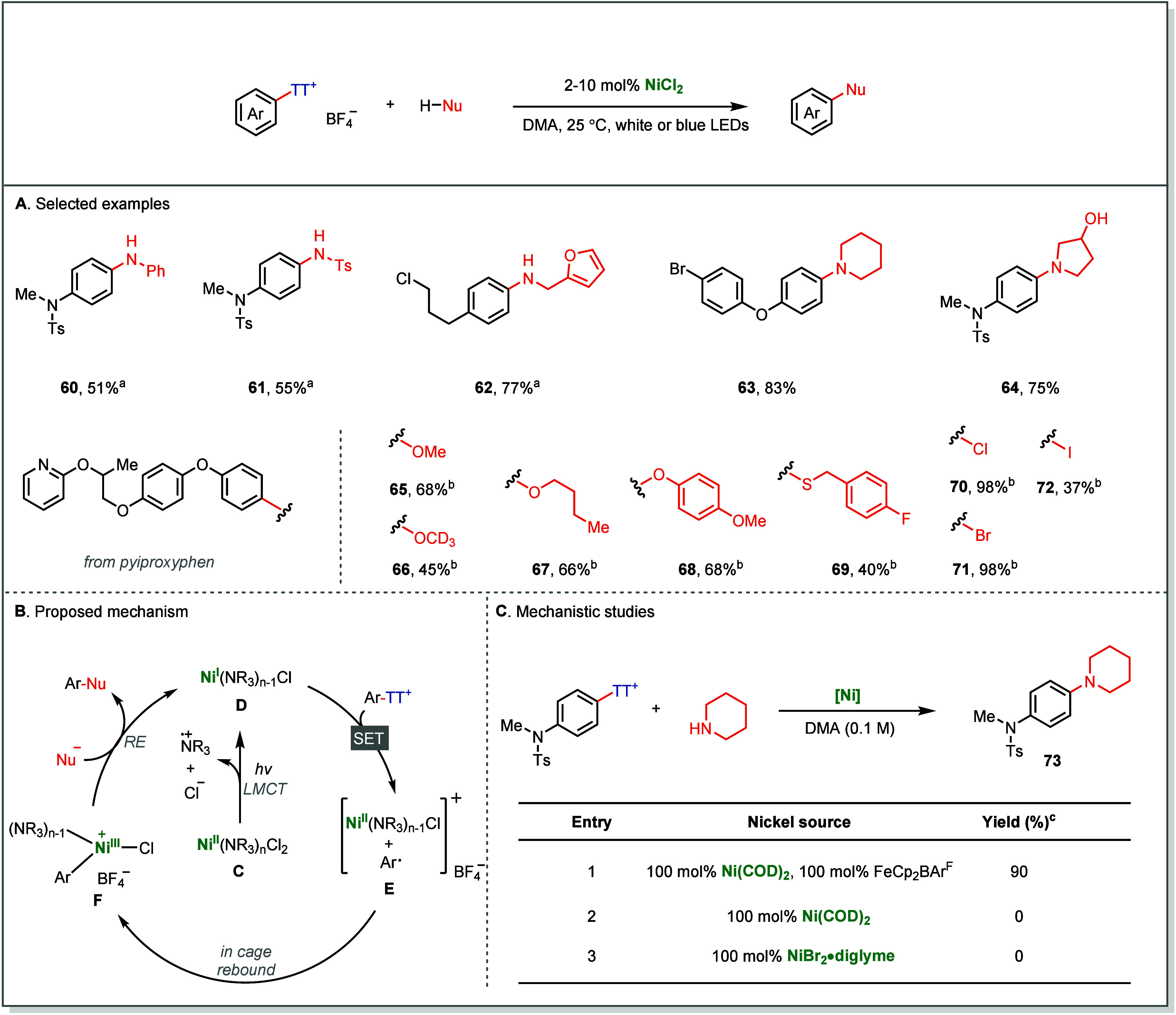
Photoinduced Ni-catalyzed late-stage C–heteroatom coupling using arylthianthrenium salts. ^*a*^With BTMG as additive. ^*b*^With quinuclidine as additive. ^*c*1^H NMR yield by using 1,3,5-trimethoxybenzene as the internal standard.

## 4 Thianthrenium Salts in EnT Catalysis

Beyond the generation of aryl radicals from aryl (pseudo)­halides through mesolytic cleavage following single-electron reduction, homolytic bond cleavage of electronically excited aryl (pseudo)­halides offers an alternative route to radical formation. Different from mesolytic scission, homolysis yields two radicals simultaneously, establishing a conceptually distinct reactivity pathway that expands the scope of radical chemistry beyond conventional SET-based mechanisms (Figure 13A). Direct excitation of aryl (pseudo)­halides to their singlet states generally requires high-energy UV–C irradiation (<280 nm), which limits their synthetic utility.^33^ A more practical approach relies on triplet energy transfer from an excited photocatalyst to substrates, which requires that the substrate’s triplet energy be comparable to or lower than that of the photosensitizer (Figure 13B). However, simple aryl halides such as phenyl bromide or chloride possess high triplet energies (*E*_T_ ≈ 78–82 kcal/mol), exceeding those of common photocatalysts (*E*_T_ < 66 kcal/mol), rendering EnT activation energetically inaccessible.^33^

**Figure 13:**
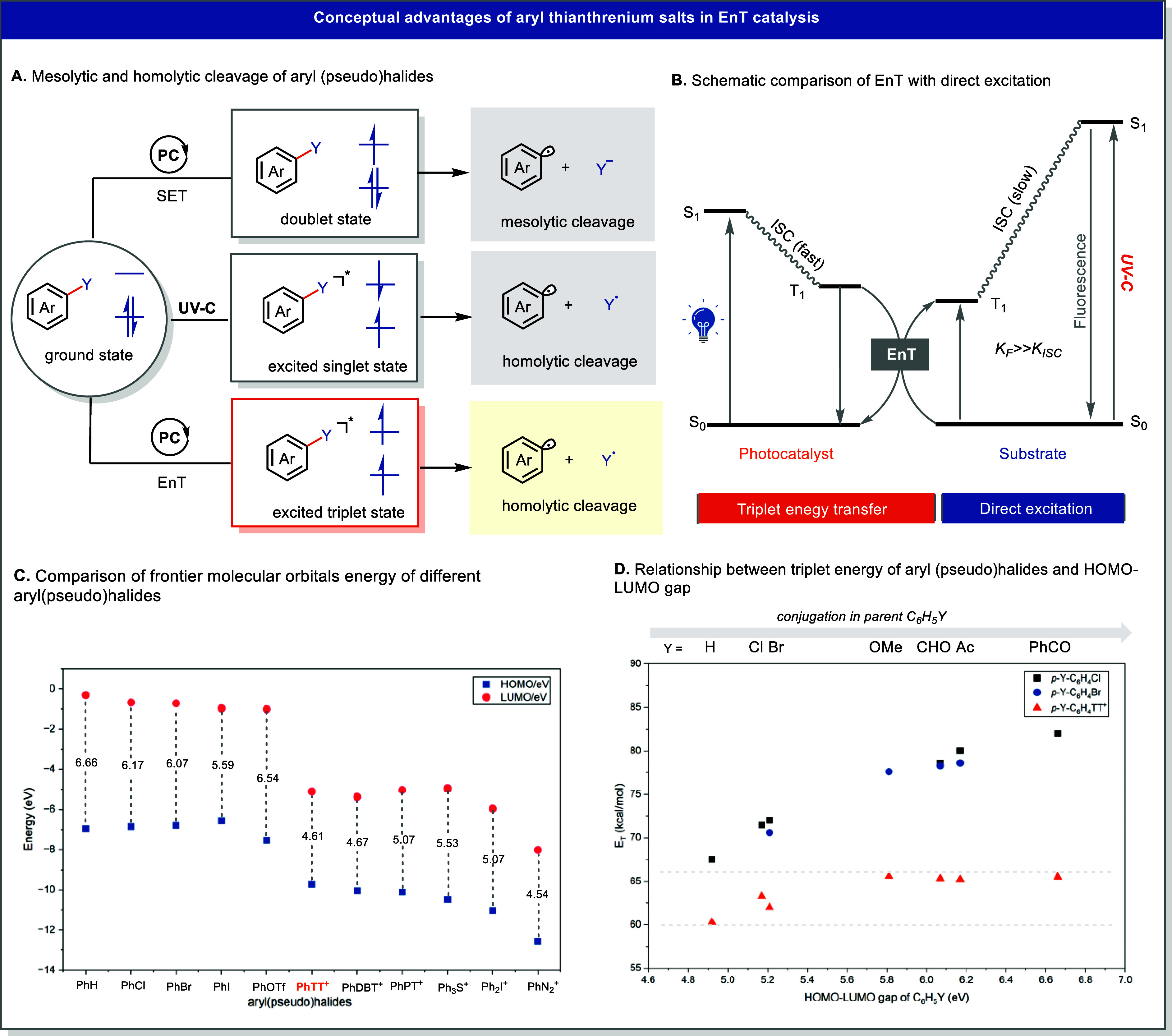
Conceptual advantages of aryl thianthrenium salts in EnT catalysis. (A) Mesolytic and homolytic cleavage of aryl (pseudo)­halides. (B) Schematic comparison of EnT with direct excitation. (C) Comparison of frontier molecular orbitals energy of different aryl­(pseudo)­halides. (D) Relationship between triplet energy of aryl (pseudo)­halides and HOMO–LUMO gap.

While our initial focus was on SET pathways, we soon recognized that thianthrenium salts could also be activated by triplet energy transfer. This discovery emerged from a simple question—could the thianthrenium group lower the triplet energy of an aryl system enough to make energy transfer, rather than electron transfer, the dominant photochemical event? Indeed, aryl thianthrenium salts exhibit triplet energies in a narrow range of 60–66 kcal/mol across diverse aryl substituents,^4^ allowing them within the operational window of typical EnT photocatalysts.^67^ To understand this distinction, DFT calculations were performed to understand the electronic structure features relevant to energy transfer reactivity. Although the bent thianthrenium framework limits π-conjugation, calculations reveal that Ph-TT^+^ possesses a substantially narrower HOMO–LUMO gap than both phenyl halides and structurally related phenyl sulfonium salts (Figure 13C). The LUMO of Ph-TT^+^ is markedly stabilized relative to neutral aryl halides, while its HOMO remains comparatively high with respect to other cationic aryl salts—an effect attributed to the electronic interplay between the positively charged and neutral sulfur atoms within the TT core. Although triplet energies cannot be directly derived from HOMO–LUMO gaps, they often follow similar trends, as both parameters reflect the degree of π-conjugation across the molecular framework (Figure 13D). As a result, the triplet energy of Ph-TT^+^ is significantly lower than that of phenyl halides (∼82 kcal/mol) or triphenyl sulfonium salts (∼75 kcal/mol), placing it well within the excitation range of visible-light EnT photosensitizers.

Experimentally, this concept was validated through EnT-mediated arylation reactions of ethylene. When the arylthianthrenium salt **74** was irradiated (390 nm) under 1 atm of ethylene in the presence of various photosensitizers, the reaction rate of product formation correlated with the triplet energy of the sensitizer rather than its redox potential, which supports an EnT rather than SET mechanism (Figure 14A).^4^ Using thioxanthone (*E*_T_ = 65.5 kcal/mol) as the sensitizer, the arylethylated thianthrenium salt **75** formed in 82% yield within 2 min. In contrast, aryl chlorides, bromides, iodides, and triflates (*E*_T_ ≈ 82 kcal/mol) remained inert under identical conditions. Other substrates with accessible triplet energies, such as biphenyl halides, displayed little reactivity, likely due to high C–X bond dissociation energies or less efficient orbital overlap with the excited sensitizer. Similarly, other sulfonium salts, including triphenyl- and dibenzothiophenium derivatives, showed negligible reactivity, possibly due to the low stability of the corresponding sulfonium radical intermediates (Figure 14B).

**Figure 14:**
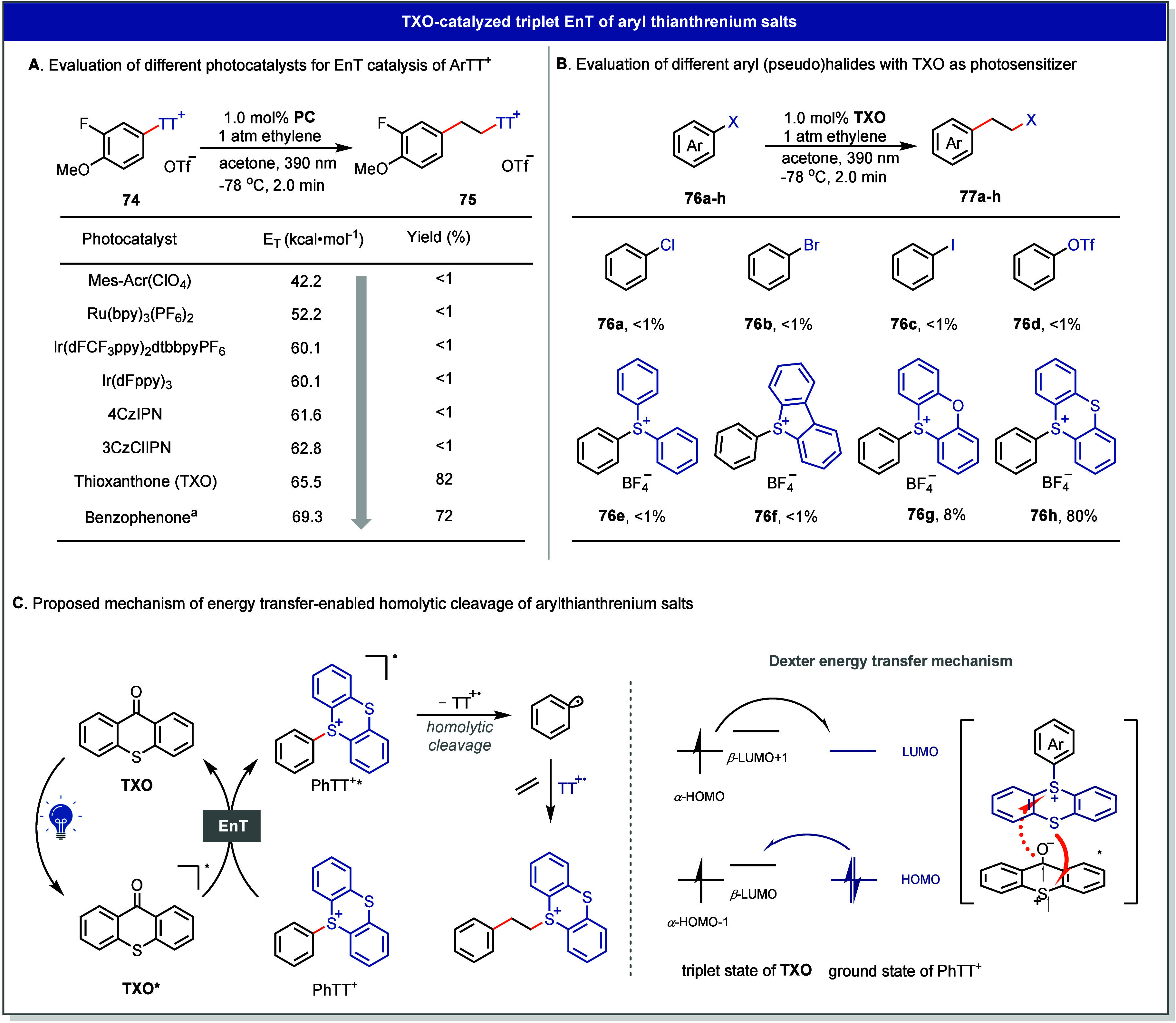
Development of EnT catalyzed aryl-hetero difunctionalization of alkenes. (A) Evaluation of different photocatalysts for EnT catalysis of Ar–TT^+^. (B) Evaluation of different aryl (pseudo)­halides with TXO as photosensitizer. (C) Proposed mechanism of energy transfer-enabled homolytic cleavage of arylthianthrenium salts. ^*a*^20 mol % catalyst loading, 20 min.

The proposed mechanism involves Dexter-type energy transfer from excited TXO to Ar-TT^+^ with a quantum yield of energy transfer up to 36%, producing triplet-excited Ar-TT^+^ that undergo rapid C–S bond homolysis to yield an aryl radical and a persistent TT^•+^ species. Selective cross-coupling of two different radicals is generally challenging, as radical recombination processes are often nearly diffusion-controlled. Generation of radicals at equal rates with significantly different lifetimes leads to a steady-state concentration imbalance, under which selective cross-coupling becomes dominant via the persistent radical effect (PRE), typically involving a persistent radical and a transient radical.^68^ As a result, the persistency of TT^•+^ is yet another distinctive feature that sets TT chemistry apart from most other substituents, such as halides and other sulfonium salts, because the radical can exist in solution, ready to accept another radical once the productive chemistry has occurred. Accordingly, the aryl radical adds to ethylene to form a transient homobenzyl radical, which subsequently recombines with TT^•+^ to afford the arylethylated product. The resulting arylethyl thianthrenium intermediates serve as versatile electrophiles for one-pot formal aryl-hetero difunctionalization of alkenes with diverse nucleophilic substitutions, furnishing β-arylethylamines, ethers, thioethers, and halides, among others (Figure 15).

**Figure 15:**
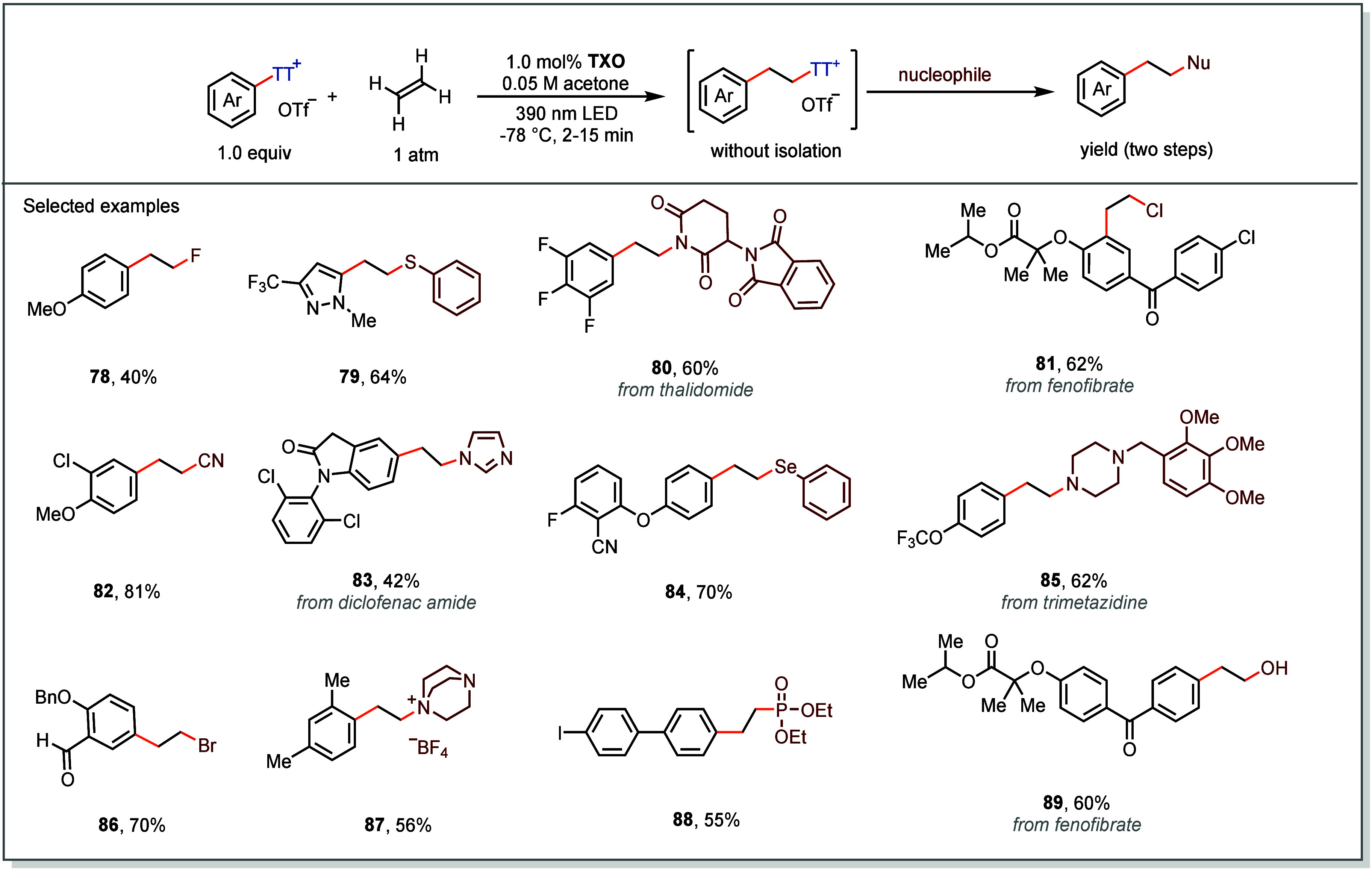
Selected substrate scope of EnT catalyzed aryl-hetero difunctionalization of alkenes.

At a conceptual level, the unusually efficient EnT from TXO to ArTTs with a quantum yield of 36% can be rationalized through orbital alignment (Figure 14C). The excited TXO features frontier orbitals (α-HOMO and β-LUMO) localized on the carbonyl carbon and sulfur atoms, which spatially coincide with the neutral and cationic sulfur orbitals of Ph-TT^+^. This geometric complementarity, which arises from their analogous tricyclic frameworks, facilitates rapid Dexter energy transfer. Other sensitizers that lack such orbital symmetry, such as benzophenone, exhibit inferior performance. Collectively, these results demonstrate that the thianthrenium framework not only lowers the triplet energy to match visible-light photosensitizers but also provides an orbital architecture optimized for efficient energy transfer. This dual advantage establishes Ar-TTs as efficient triplet energy acceptors, enabling homolytic activation under mild conditions with broad functional-group tolerance, which has not yet been accessible to conventional aryl (pseudo)­halides.

## 5 Thianthrenium Salts in Direct Visible-Light Induced Homolysis

While most aryl and alkyl thianthrenium salts do not absorb visible light and require UV irradiation for direct homolytic cleavage,^40^ the CF_3_–TT^+^ salt represents an exception. Despite its weak absorption in the visible region, its low C–S bond dissociation energy enables direct photolysis under visible-light irradiation.^35^ The resulting CF_3_ radical readily engages with heteroarenes, [1.1.1]­propellane, and alkenes, facilitating diverse trifluoromethylation reactions (Figure 16).^35–37^ Meanwhile, the thianthrene radical cation serves either as an internal oxidant^35^ or participates in the formation of product^37^ or intermediate.^36^

**Figure 16:**
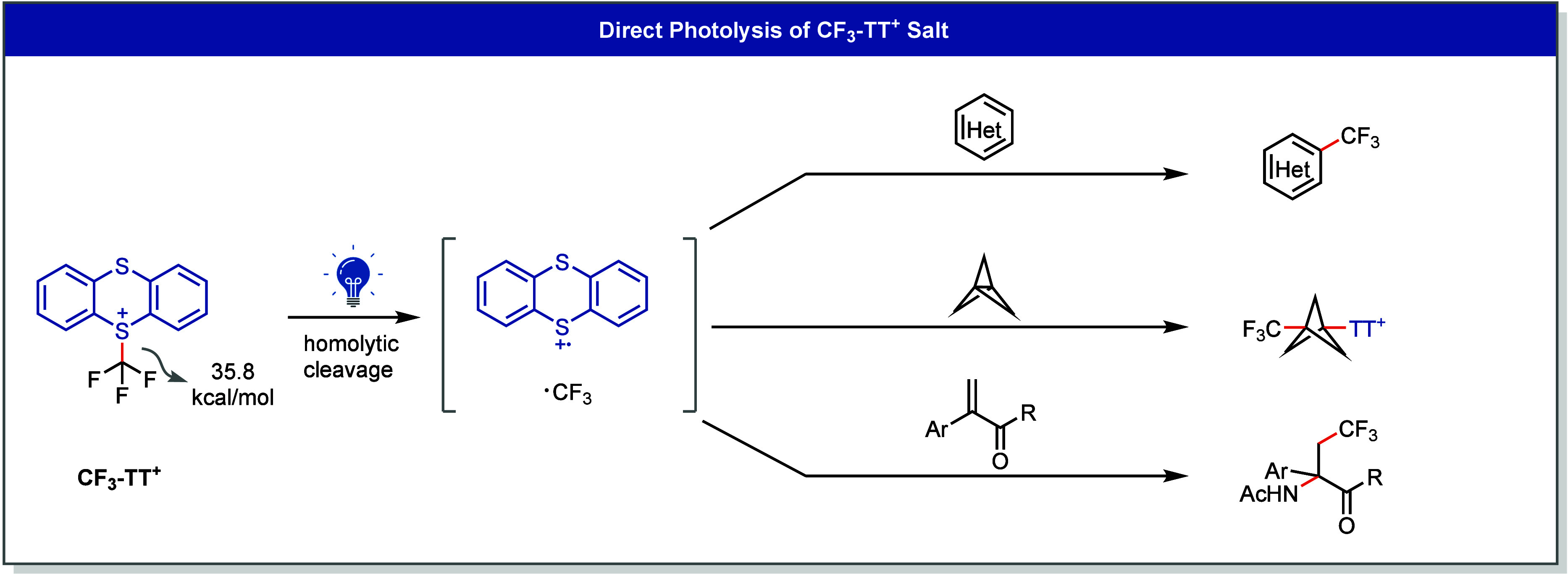
Trifluoromethyl thianthrenium salts in direct photolysis.

A more general radical generation under direct photolysis was realized when a rationally designed selenoxide was introduced to site-selective C–H functionalization of tyrosine residues of peptides and proteins in aqueous media.^4^ Like their TT analogues, the LUMO of the resulting selenonium salt possesses a large contribution from the antibonding orbital of C–Se σ bond. Notably, these salts exhibit absorption in the 365–465 nm range and feature relatively low C–Se BDEs of ∼71 kcal/mol. The resulting aryl radicals participate in a variety of synthetically useful transformations, including coumarin annulation (**90**), bromination (**91**), and iodination (**92**) and allow for single-atom modifications of peptides and proteins with high site selectivity (Figure 17A).

**Figure 17:**
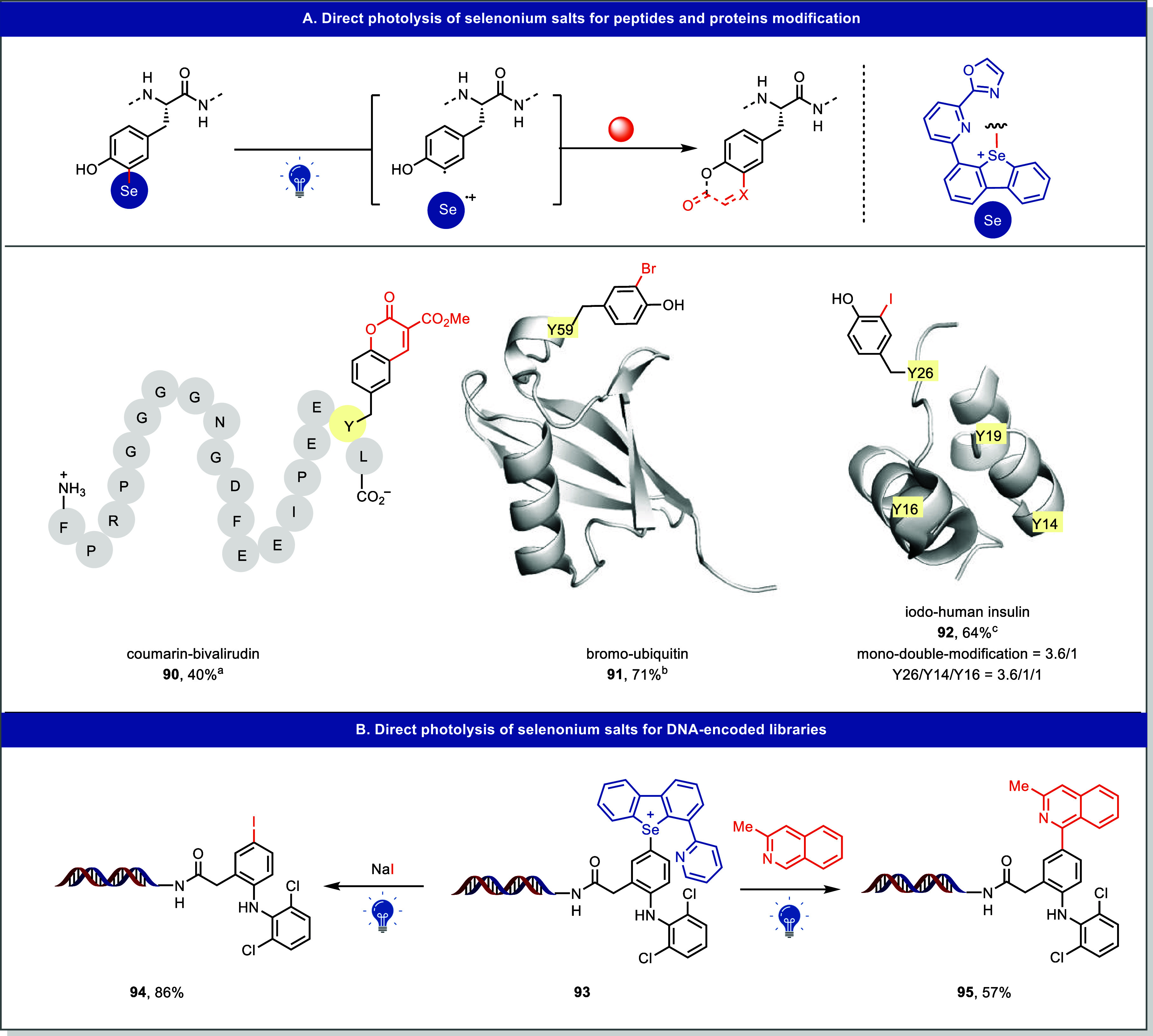
Direct photolysis of selenonium salts for biomacromolecules modification. (A) Direct photolysis of selenonium salts for peptides and proteins modification. (B) Direct photolysis of selenonium salts for DNA-encoded libraries. ^*a*^FeSO_4_·7H_2_O, dimethyl 2-(methoxymethylene)­malonate, then Na-glycine buffer (pH 9.0), 390 nm LEDs for 10–20 min. ^*b*^CuBr_2_, Cu­(MeCN)_4_PF_6_, KBr, 390 nm LEDs for 10–20 min. ^*c*^NaI, *i*PrI, 390 nm LEDs for 10–20 min.

This platform was further extended to on-DNA C–H functionalization of electron-rich arenes.^42^ Upon visible-light activation, the corresponding DNA-tethered aryl radicals were generated and successfully participated in follow-up reactions such as iodination (**94**) and Minisci arylation (**95**), demonstrating the compatibility of direct photolysis-based radical generation with DNA-encoded library synthesis (Figure 17B).

## 7 Conclusion and Future Outlook

Thianthrenium salts have emerged as versatile reagents for radical generation through diverse activation modes, including photoredox catalysis, energy transfer catalysis, and direct photolysis. Their unique combination of high reduction potentials, low triplet energy, and rapid C–S bond cleavage enables radical formation under visible-light conditions. Recent advances have extended their use to late-stage functionalization of biomacromolecules. Despite the broad utility of thianthrenium salts in photochemistry, several challenges remain: (1) *High loadings of TM catalyst*: In TM/photoredox dual catalysis, relatively high loadings of TM catalysts are often required to ensure sufficient reactivity and suppress undesired side reactions. Rational ligand design may improve catalyst efficiency, enabling lower loadings while maintaining selectivity and turnover. (2) *Low atom economy*: Current protocols generate stoichiometric amounts of thianthrene as a byproduct. The development of catalytic TT turnover systems under photoredox conditions could significantly improve sustainability. (3) *Selenylation under harsh conditions*: Site-selective C–H selenylation of biomacromolecules requires acidic conditions (pH = 3) with current selenoxides, which limits compatibility with sensitive peptides and proteins. Further rational design could expand the scope of biocompatible selenoxide platforms that operate under milder, physiologically relevant conditions.
